# Dr. Alfred H. Robbins

**Published:** 1907-01

**Authors:** 


Dr. Alfred H. Robbins, of Rochester, Ind., died at his home, October 8, 1906, aged 80 years. He graduated at the University of Buffalo in 1850, had practised over fifty years at Rochester, and had represented his county in the state legislature for three terms.

